# Adaptive correction method of hybrid aberrations in Fourier ptychographic microscopy

**DOI:** 10.1117/1.JBO.28.3.036006

**Published:** 2023-03-13

**Authors:** Ruofei Wu, Jiaxiong Luo, Jiancong Li, Hanbao Chen, Junrui Zhen, Sicong Zhu, Zicong Luo, Yanxiong Wu

**Affiliations:** a Foshan University, School of Physics and Optoelectronic Engineering, Foshan, China; b Ji Hua Laboratory, Foshan, China

**Keywords:** Fourier ptychographic microscopy, aberration correction, phase recovery, Zernike polynomials

## Abstract

**Significance:**

Fourier ptychographic microscopy (FPM) enables quantitative phase imaging with a large field-of-view and high resolution by acquiring a series of low-resolution intensity images corresponding to different spatial frequencies stitched together in the Fourier domain. However, the presence of various aberrations in an imaging system can significantly degrade the quality of reconstruction results. The imaging performance and efficiency of the existing embedded optical pupil function recovery (EPRY-FPM) aberration correction algorithm are low due to the optimization strategy.

**Aim:**

An aberration correction method (AA-P algorithm) based on an improved phase recovery strategy is proposed to improve the reconstruction image quality.

**Approach:**

This algorithm uses adaptive modulation factors, which are added while updating iterations to optimize the spectral function and optical pupil function updates of the samples, respectively. The effectiveness of the proposed algorithm is verified through simulations and experiments using an open-source biological sample dataset.

**Results:**

Experimental results show that the proposed AA-P algorithm in an optical system with hybrid aberrations, recovered complex amplitude images with clearer contours and higher phase contrast. The image reconstruction quality was improved by 82.6% when compared with the EPRY-FPM algorithm.

**Conclusions:**

The proposed AA-P algorithm can reconstruct better results with faster convergence, and the recovered optical pupil function can better characterize the aberration of the imaging system. Thus, our method is expected to reduce the strict requirements of wavefront aberration for the current FPM.

## 1 Introduction

High resolution (HR), large field of view (FOV), and quantitative phase imaging have long been the goals of optical microscopy. However, conventional microscopy struggles to combine these objectives, which significantly limits its practical application.^1^^,^^2^ Fourier ptychographic microscopy (FPM) is a representative new computational imaging technique that employs angularly varying illumination to obtain a series of low-resolution (LR) images corresponding to different spatial frequencies and frequency domain stitching to broaden the spectrum of the sample, computationally breaking the diffraction limit of the objective lens.^3^ The technique combines the principles of phase recovery^4^^,^^5^ and synthetic aperture^6^^,^^7^ to recover the intensity and phase information of a sample under a low numerical aperture (NA) objective with a large FOV, increasing the spatial bandwidth product of the microscope by at least an order of magnitude.^8^ However, there are several inevitable imaging process aberrations far from the edge of the central FOV, such as defocus aberrations caused by the uneven thickness and inaccurate focus of the sample, spherical and coma aberrations of the microscope objective, and the image dispersion of the off-axis LED beam refracted by the lens, which cannot be focused at one point.^9^^–^^11^ A hybrid of these aberrations has serious effects on the reconstruction quality of the FPM.

The FPM proposed in 2013 by Zheng et al. has received extensive attention and is researched worldwide.^3^ FPM reconstruction frameworks combined with correction methods have been proposed to further optimize the performance of FPM and reduce the impact of wavefront aberrations on the quality of FPM reconstruction. The existing optimization schemes fall into two main categories: one is to pre-estimate the imaging system aberrations and correct them in the phase recovery algorithm, while the other is to optimize the reconstruction algorithm directly to correct the imaging system aberrations without pre-estimation. To estimate the imaging system aberrations in advance, Zheng et al.^9^ proposed a generalized pattern search algorithm based on phase retrieval to measure aberration coefficients, followed by aberration correction of the acquired images using a deconvolution method, which was highly sensitive to component movement in the imaging system. It inaccurately estimated higher-order aberrations and had a high computational cost. Furthermore, attempts to combine the aberration correction algorithm with the reconstruction algorithm were made. Ou et al. proposed an embedded optical pupil function recovery algorithm (EPRY-FPM),^10^ which could recover the optical pupil function of the imaging system using the acquired LR image dataset alone without estimating the imaging system aberration in advance. EPRY-FPM can better correct aberrations, and it is currently considered a mainstream method. However, the recovery results were not satisfactory when hybrid aberrations were encountered owing to the influence of spectrum and pupil function update strategy because EPRY-FPM used the traditional FPM reconstruction framework, which was combined with an alternating iterative reconstruction algorithm (AP algorithm).^3^ Subsequently, some researchers used deep learning methods to optimize aberrations. Sun et al. proposed a neural network framework with optical pupil function recovery,^12^ where the complex amplitude image of a sample was obtained by minimizing the loss function. However, the framework did not consider the effect of spatially varying aberrations, and its generalization was low considering the requirement of a long time to retrain for different samples and the high demand for hardware processing. Therefore, the development of a correction method for the existing FPM imaging system that does not increase the computational complexity has a fast convergence rate, and is more robust to aberrations is necessary.

An AA algorithm was used in our previous study to optimize the spectral update strategy of the reconstruction algorithm for an improved FPM reconstruction framework.^13^ The algorithm improved the convergence speed of the reconstruction algorithm and its robustness to noise, but the impact of various potential imaging system aberrations on the reconstruction quality was not considered. Therefore, this study proposes an FPM aberration correction reconstruction algorithm (AA-P) based on an improved phase recovery strategy. The AA-P optimizes the updating process of the sample spectral and optical pupil functions by adaptively selecting a modulation factor from the acquired LR image dataset. Compared to conventional aberration correction methods such as EPRY-FPM, AA-P can reconstruct better results with faster convergence, and the recovered optical pupil function can better characterize the imaging system aberrations. Furthermore, the effectiveness and advantages of the AA-P are verified through simulations and experiments on biological sample datasets.

The remainder of this paper is organized as follows. Section 2 briefly outlines the basic model of the conventional FPM, the effect of aberrations in an optical system on the FPM, and the specific procedure of the AA-P proposed in this study. Section 3 verifies the effectiveness of the AA-P through simulations and real experiments, while Sec. 4 summarizes and discusses the results of this study.

## 2 Materials and Methods

### 2.1 FPM Forward Model

**Fig. 1:**
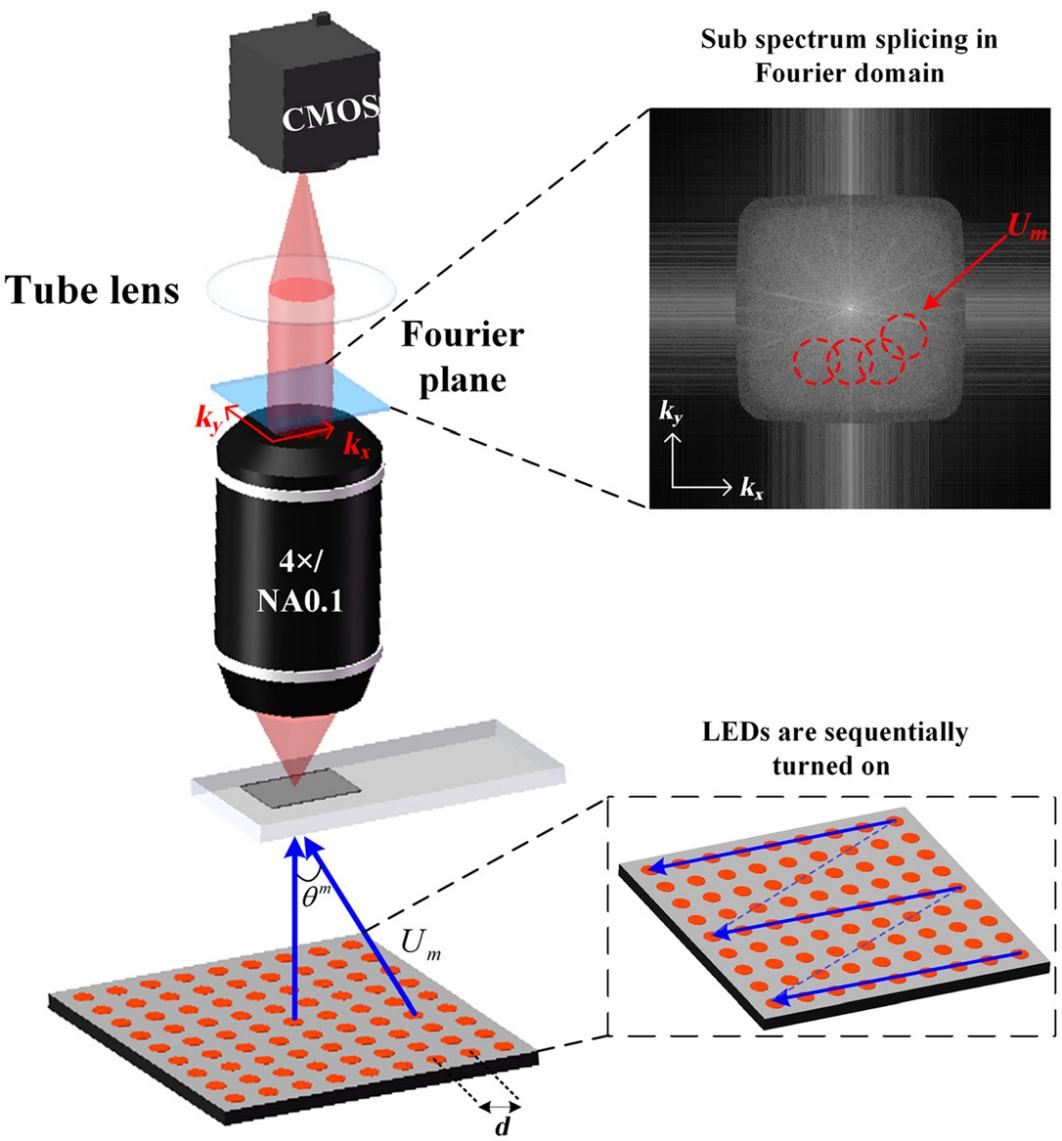
Typical FPM model and imaging process.

Figure 1 shows the model and imaging process of a typical FPM system. The model mainly consists of a programmable LED array with a low NA objective (4×/0.1 NA objective is used as an example in this study) for a general microscopic imaging system. The imaging process of FPM includes image acquisition and image reconstruction. During image acquisition, the spectral information of the sample beyond the bandwidth of the objective lens is moved within the NA of the objective lens and positioned on the back focal plane of the objective lens (i.e., the spectral plane of the objective lens) by sequentially lighting the LED array (shown in the lower right corner of Fig. 1) to provide angularly varying plane waves for illumination. As shown in the upper right corner of Fig. 1, the dashed circles are: several of the sub-spectral information corresponding to samples illuminated by plane waves of different angles, while the corresponding LR images are captured at the camera port, after which the completed LR image dataset is iteratively reconstructed using a phase recovery algorithm.^14^

The conventional microscopic illumination in the FPM image acquisition process is replaced by an array of programmable LEDs. Each lit LED provides an illumination sample of wave vector $U_{m}$, which varies with an angle θ. $U_{m}$ for the m’th LED can be expressed as $U_{m}=(\frac{\sin\;\theta_{x}^{\left(m\right)}}{\lambda },\frac{\sin\;\theta_{y}^{\left(m\right)}}{\lambda })$, where λ denotes the illumination wavelength of light in air and $\left(\theta_{x}^{\left(m\right)},\theta_{y}^{\left(m\right)}\right)$ denote the angle of incidence of the m’th LED. The image sensor (CMOS) is limited by a sampling rate. Hence, to only capture the light wave intensity, the captured intensity image can be expressed as

$$I_{m}(r)={\left|F^{-1}\{O(u-U_{m})\cdot P(u)\}\right|}^{2},$$ (1)

where r=(x,y) represents the coordinates in a two-dimensional space domain, which signifies the position of each LED unit in the array; $u=(k_{x},k_{y})$ represents the spatial coordinates in the two-dimensional Fourier domain; $O(u-U_{m})$ represents the spectrum of the sample under illumination corresponding to the m’th LED; and F and $F^{-1}$ represent the Fourier transform and inverse transform, respectively. P(u) represents the optical pupil function of the imaging system, which is regarded as the coherence transfer function in the FPM system, and is regarded as an ideal circular domain function without aberration in the ordinary FPM algorithm. Specifically, it can be expressed as

$$P(u)=|P(k_{x},k_{y})|=\{\begin{array}{cc} 1, & (k_{x}^{2}+k_{y}^{2})\leq {\left(\frac{NA}{\lambda }\right)}^{2}\\ 0, & \text{otherwise} \end{array},$$ (2)

where NA is the numerical aperture of the objective lens and $\left|P(k_{x},k_{y})\right|$ denotes the amplitude of the optical pupil function. According to Eqs. (1) and (2), P(u) corresponds to the low-pass filtering of light field information of a sample when a LED unit irradiates the sample through the FPM imaging system. Thus, a series of LR images captured during image acquisition represent the subspectral information of the sample in different spectral regions. The captured LR images are used as the null domain amplitude constraint, while P(u) is used as the frequency constraint in the Fourier domain to expand the spectral range of the sample by stitching.

### 2.2 Aberrations in FPM Imaging Systems and Their Effects

The forward imaging model of FPM in an ideal aberration-free case was discussed in Sec. 2.1; however, in practical experimental platforms and applications, various aberrations are inevitably introduced into the imaging system. It is well known that linear proportionality exists between aberration effect and imaging FOV,^10^ which is especially important for FPM imaging systems with a large FOV and HR imaging. Since aberrations are present and most microscope objectives are in circular domains with symmetric rotational properties, the optical pupil function of the microscope objective can no longer be expressed by Eq. (2). Hence, P(u) is expressed as^11^

$$P(u)=|P(k_{x},k_{y})|\exp[i2\pi \overset{15}{\underset{j=1}{\sum }}a_{j}Z_{j}(\rho ,\theta )],$$ (3)

where $\left|P(k_{x},k_{y})\right|$ is the amplitude of the light pupil function shown in Eq. (2), $Z_{j}(\rho ,\theta )$ and $a_{j}$ are the Zernike polynomials and their coefficients corresponding to the aberration, respectively.

**Fig. 2:**
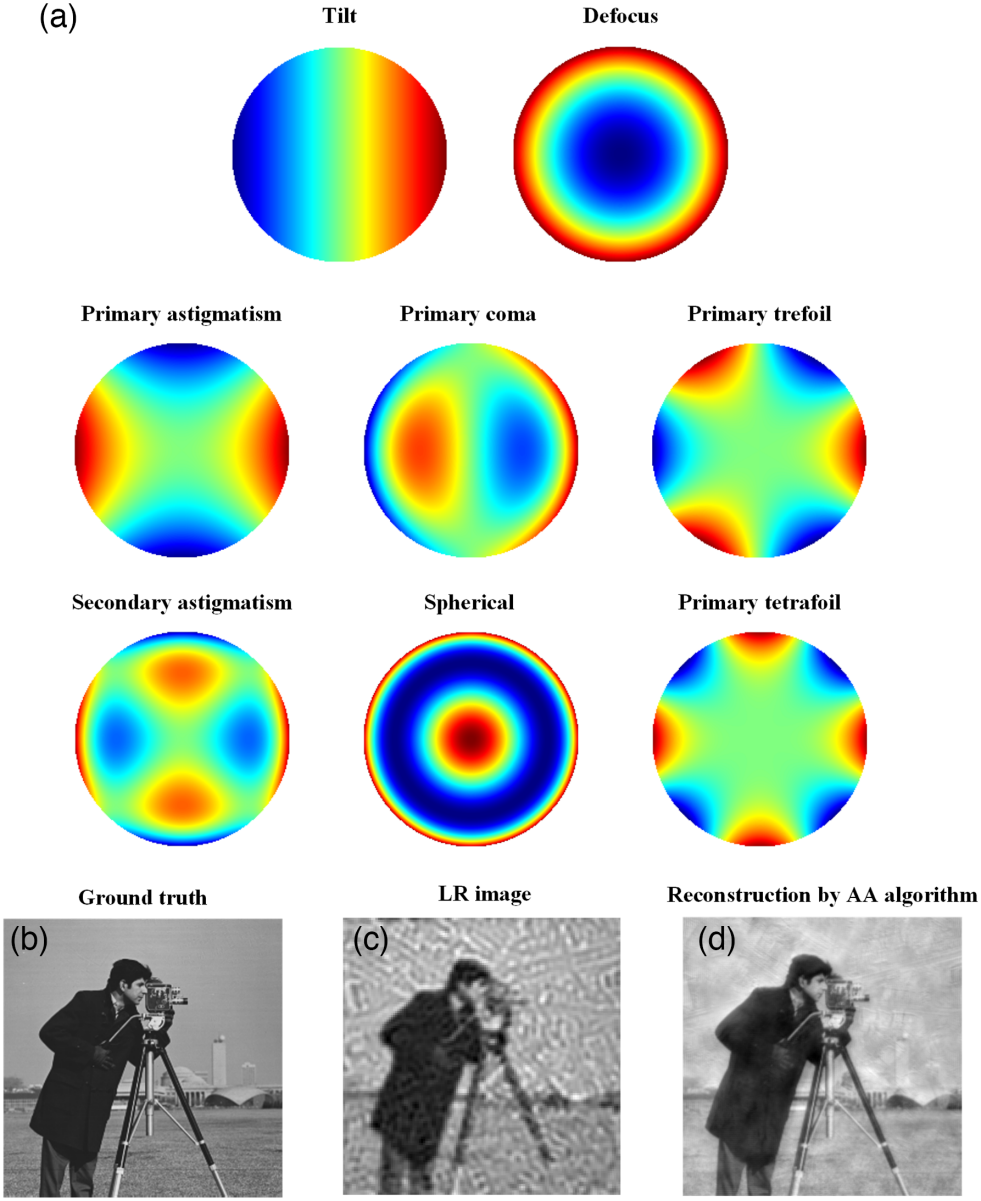
Several potential aberrations of the microscope objective and the effect of the presence of hybrid aberrations on the FPM reconstruction quality. (a) Two-dimensional display of several aberrations of the first 15 Zernike polynomial coefficients: tilt, defocus, primary astigmatism, primary coma, primary trefoil, secondary astigmatism, spherical aberration, and primary trefoil aberration. (b)–(d) Reconstruction results of (b) the original HR input image, (c) acquired LR image, and (d) the image obtained using the AA algorithm in the simulated experiment.

A real FPM imaging system has usually several types of aberrations, such as: tilt, defocus, spherical, primary and secondary astigmatism, primary coma, primary trefoil, primary tetrafoil, etc. If they exist simultaneously, they are called hybrid aberrations. In this study, we simulate the above types of aberrations using the first 15 terms of the Zernike polynomial, as shown in Table 1, where ρ(0≤ρ≤1) is the radial distance and θ(0≤θ≤2π) is the azimuthal angle, and both variables are expressed in polar coordinates. Figure 2(a) shows a two-dimensional presentation corresponding to each aberration type in Table 1. In this study, each coefficient is simulated using ±(0−0.2) values in the simulation of hybrid aberrations.^12^^,^^15^^,^^16^

**Table 1:** First 15 terms of Zernike and the assumed coefficients of its simulation.

Items	Zernike polynomial	Aberration
1	1	Constant term
2	ρ cos θ	Tilt x
3	ρ sin θ	Tilt y
4	$2\rho^{2}-1$	Defocus
5	$\rho^{2}\;\sin\;2\theta$	Primary astigmatism x
6	$\rho^{2}\;\cos\;2\theta$	Primary astigmatism y
7	$(3\rho^{2}-2)\rho \;\sin\;\theta$	Primary coma x
8	$(3\rho^{2}-2)\rho \;\cos\;\theta$	Primary coma y
9	$\rho^{3}\;\cos\;3\theta$	Primary trefoil x
10	$\rho^{3}\;\sin\;3\theta$	Primary trefoil y
11	$6\rho^{4}-6\rho^{2}+1$	Primary spherical
12	$(4\rho^{2}-3)\rho^{2}\;\cos\;2\theta$	Secondary astigmatism x
13	$(4\rho^{2}-3)\rho^{2}\;\sin\;2\theta$	Secondary astigmatism y
14	$\rho^{4}\;\cos\;4\theta$	Primary tetrafoil x
15	$\rho^{4}\;\sin\;4\theta$	Primary tetrafoil y

Figures 2(b)–2(d) illustrate the effects of hybrid aberrations on the captured LR images and reconstruction quality. The captured LR images showed several streaks because of the pupil aberration of the objective, as shown in Fig. 2(c). The appearance of phase residuals in Eq. (3) affected the spectral distribution of the sample to some extent, which eventually affected the reconstruction quality (here, the reconstruction is performed using AA algorithm, which has been proven to be generally stable and advantageous for FPM reconstruction), as shown in Fig. 2(d).^13^ Subsequent corrections and comparisons of various algorithms are further discussed in Sec. 3.1. Therefore, it is necessary to improve the robustness and quality of the FPM reconstruction algorithm for hybrid aberrations conditions.

### 2.3 AA-P Aberration Correction Algorithm Reconstruction Framework

**Fig. 3:**
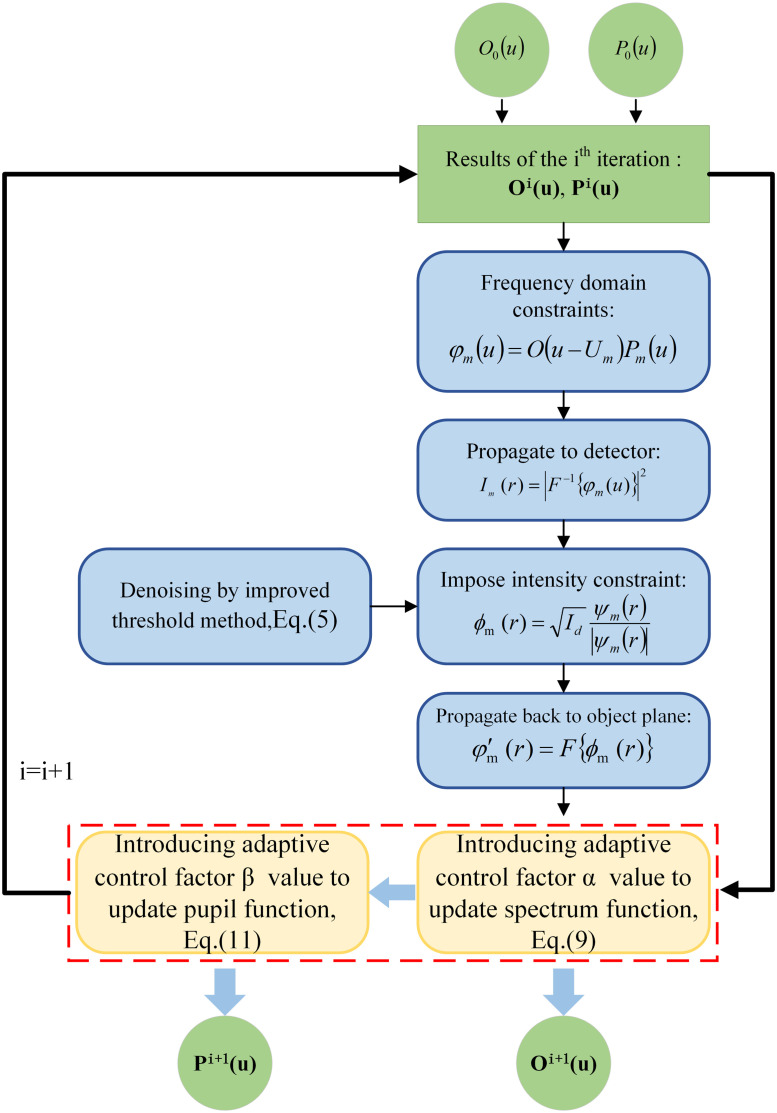
Flowchart of the AA-P algorithm.

Based on the potential aberrations of the FPM imaging model and system, an FPM forward imaging model using the AA algorithm was implemented in this study. However, the reconstruction framework of the AA algorithm set the pupil function to an ideal value and the inaccuracy of the previously fixed pupil function could seriously degrade the reconstruction quality when hybrid aberrations occurred in the system. Therefore, the AA-P algorithm is proposed. The specific process and steps are discussed further, as shown in Fig. 3.

Step 1: Initially, the sample spectrum and light pupil function of the imaging system are assumed. The Fourier variation of the LR image corresponding to the positive incidence of the LED array center is taken as the initial HR spectrum, which is marked as $O_{0}(u)$ and the light pupil function is marked as $P_{0}(u)$. The initial assumption does not need to consider the aberration, and the circular domain function of Eq. (2) can be used. However, the sample spectrum and light pupil function are updated in subsequent iterations, and $O^{i}(u)$ and $P^{i}(u)$ are recorded as the sample spectrum and light pupil function of the i’th iteration.
Step 2: A frequency domain constraint is determined using the frequency-domain interception process, where $P_{m}(u)$ is used to intercept the sample HR spectrum after the completion of the last iteration. The intercepted subspectral region corresponding to the frequency domain of the LR image acquired by the LED unit irradiating the sample can be expressed as $\phi_{m}(u)=O(u-U_{m})\cdot P_{m}(u)$, where m is expressed as the LR image index, which is Fourier inverse transformed to obtain the image acquired by the digital detector, given by $I_{m}(r)={\left|F^{-1}\{\phi_{m}(u)\}\right|}^{2}$.
Step 3: Denoising process and intensity constraint: An average intensity difference between the actual acquired image $I_{m}(r)$ and the target image $I_{t}(r)$ is calculated to determine the threshold value, $Threshold_{m}=\langle I_{m}(r)\rangle -\langle I_{t}(r)\rangle$, which is used for noise reduction on the LR image as^17^
  $$I_{d}(r)=I_{m}(r)-{\text{Threshold}}_{m},$$ (4)
  where $I_{d}(r)$ is the noise-reduced image. Defining the inverse Fourier transform of the intercepted subspectral region in step 2 as: $\psi_{m}(r)=F^{-1}\{\phi_{m}(u)\}$, the noise-reduced LR image dataset is used to replace the amplitude of the target complex amplitude information as
  $$\varphi_{m}(r)=\sqrt{I_{d}}\frac{\psi_{m}(r)}{\left|\psi_{m}(r)\right|}.$$ (5)
  Subsequently, the spectrum of the target complex amplitude image after replacing the amplitude is obtained using the Fourier transform as $\phi_{m}^{\prime }(r)=F\{\varphi_{m}(r)\}$.
Step 4: The target spectrum and optical pupil functions are updated. To optimize the updated target spectral function, an adaptive modulation factor α is introduced and calculated as
  $$\tau =\frac{\overset{M}{\underset{m=1}{\sum }}{\text{Threshold}}_{m}}{M},$$ (6)
  $$\alpha =2({\left|P_{m}(u+U_{m})\right|}_{\max})-\tau ,$$ (7)
  where M is the total number of acquired LR images, m is the LR image index, and τ is the average arithmetic threshold of the LR image dataset. Additionally, considering the interaction between the spectral function of the sample and the light pupil function, a ratio of the current value to the maximum value of the light pupil function is set to accurately estimate the aberrations existing in the imaging system and to prevent the effects of sudden changes in the light pupil function. The ratio is calculated as
  $$W_{1}=\frac{\left|P_{m}(u+U_{m})\right|}{{\left|P_{m}(u+U_{m})\right|}_{\max}}.$$ (8)

Thus, the spectrum function is updated as

$$S_{m+1}(u)=S_{m}(u)+(\alpha -W_{1})\frac{P_{m}^{*}(u+U_{m})}{|P_{m}(u+U_{m}){|}^{2}+\zeta_{1}}[\phi_{m}^{\prime }(u)-\phi_{m}(u)],$$ (9)

where $\zeta_{1}$ is the regularization parameter, set to $\zeta_{1}=1$ for updating sample spectrum function, and “*” denotes the complex conjugate operation. α optimizes the process of spectrum updation to obtain better reconstruction quality and convergence speed, which is also used to update the sample spectrum of the AA algorithm. The optical pupil function in the previous case was kept constant throughout the iterative process, and the HR spectral information of the sample target subaperture was updated using the spectral difference before and after the intensity constraint. Similarly, the optical pupil function in this study is updated considering the aberrations present in the imaging system as

$$W_{2}=\frac{\left|S_{m}(u-U_{m})\right|}{{\left|S_{m}(u-U_{m})\right|}_{\max}},$$ (10)

$$P_{m+1}(u)=P_{m}(u)+(\beta -W_{2})\frac{\left|S_{m}^{*}(u-U_{m})\right|}{{\left|S_{m}(u-U_{m})\right|}^{2}+\zeta_{2}}[\phi_{m}^{\prime }(u)-\phi_{m}(u)],$$ (11)

where $\zeta_{2}$ is set in the same manner as $\zeta_{1}$. β is a constant used for optimizing the updated light pupil function. To obtain a better correction effect, β=α in this study. Thus, the updating principles of the optical pupil function and the sample spectrum function are basically the same.

Step 5: To update iterations for all LR images, steps 2–4 are repeated. The optical pupil function within all subapertures is guided with the sample spectral information for completing one iteration of the update.
Step 6: Steps 2–5 are repeated and subsequent iterations are continuously completed until the termination iteration condition of the algorithm is satisfied.

## 3 Experiments and Results

### 3.1 Simulation and Results

**Fig. 4:**
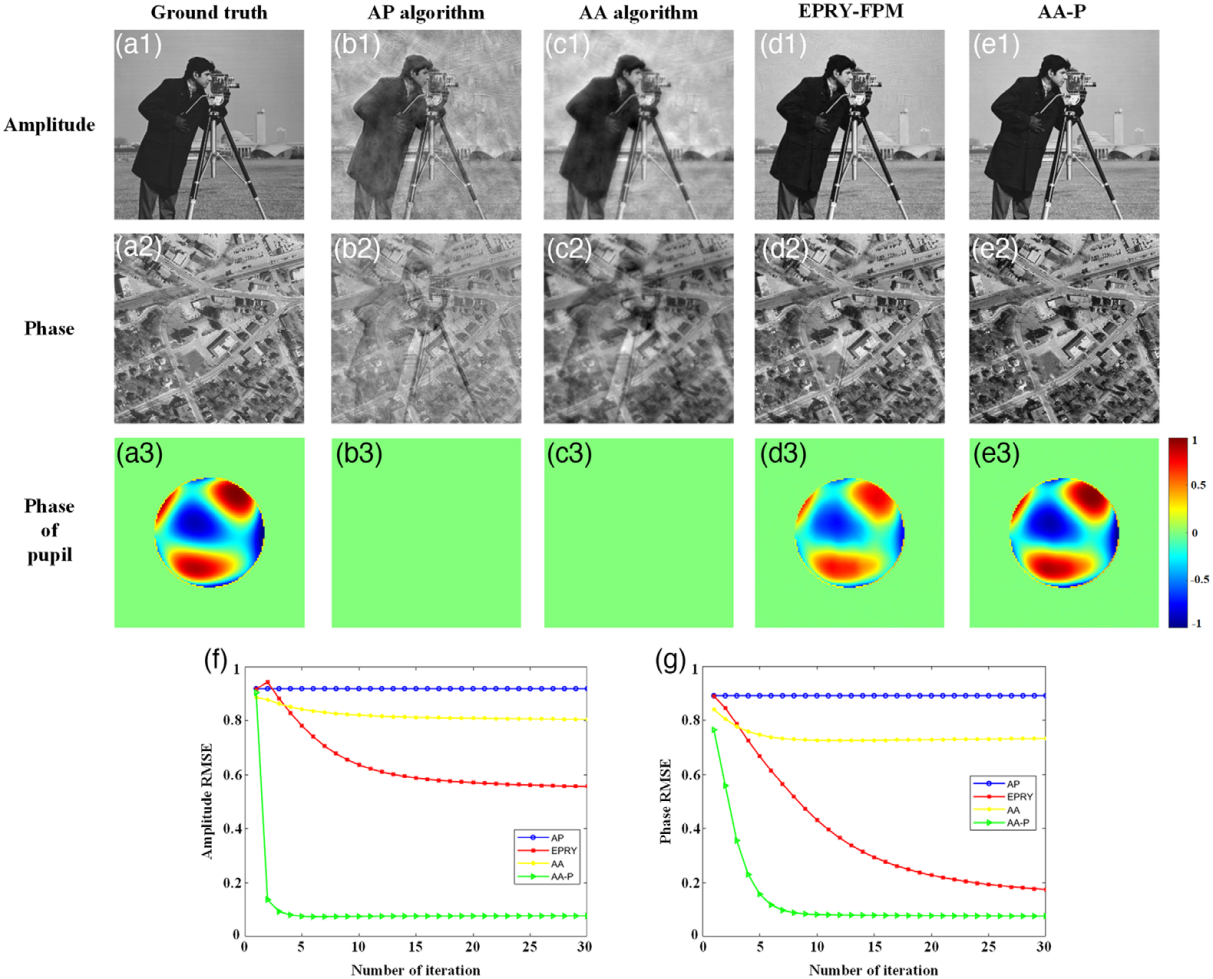
Comparison of simulated reconstruction results of different methods. (a1) Original input HR amplitude and (a2) phase images. (a3) Actual presence of hybrid aberration added to the imaging system via simulation. (b)–(e) Amplitude, phase, and recovered phase of the pupil function reconstructed by the AP, AA, EPRY-FPM, and AA-P algorithms, respectively. (f) and (g) The RMSE curves of HR amplitude and phase images, respectively, reconstructed by the four methods under the with-iteration condition.

To quantitatively analyze the effectiveness of the proposed AA-P algorithm for aberration correction and improving the quality of FPM reconstruction, the algorithm was first verified through a simulation, where the simulation parameters were consistent with the parameters of the subsequent experimental platform. For LED illumination, a 15×15 green LED array placed 90.88 mm directly below the sample, having a 4-mm spacing between adjacent LED units was simulated with an incident wavelength of 531 nm. The simulated microscope objective was set to 4× magnification, while the NA was set to 0.1, and the pixel size of the camera was 1.845  μm. “Cameraman” and “Westconcordorthophoto” of size 512×512  pixels were used as the input HR amplitude and phase images, respectively, as shown in Figs. 4(a1) and 4(a2). Therefore, the synthesized ${\mathrm{NA}}_{\mathrm{syn}}$ is 0.49. In an actual optical system, it is not only affected by a single aberration but by a variety of hybrid aberrations. In this study, the first 15 Zernike polynomial coefficients are used to simulate the hybrid aberration of the imaging system with reference to the ideas in Sec. 2.2, as shown in Fig. 4(a3).

The simulation generated 225 LR images with an overlap rate of 68.04% in the Fourier domain, and the convergence of all algorithms was guaranteed without any aberration.^18^ Then, four FPM reconstruction algorithms, AP, AA, EPRY-FPM, and the proposed AA-P, were iteratively reconstructed and compared in the simulated FPM imaging system with wavefront aberration, as shown in Fig. 4. The four algorithms were grouped into two categories, where the AP and AA algorithms were without an aberration correction function, while the EPRY-FPM and the proposed AA-P algorithms were with an aberration correction function. As shown in Figs. 4(b1) and 4(b2), the AP algorithm has the worst reconstruction quality and failed to perform normal convergence for multiple hybrid aberrations. Comparatively, the AA algorithm had an improved the reconstruction quality, owing to its superior performance, as shown in Figs. 4(c1) and 4(c2). However, the reconstruction quality of the AA algorithm was still unsatisfactory because the wavefront aberration repeatedly affected the reconstructed spectrum distribution, leading to a certain amount of crosstalk in the reconstructed intensity and phase information. The EPRY-FPM algorithm further improved the reconstruction quality, as shown in Figs. 4(d1) and 4(d2); however, it continued to suffer from the case of detail blurring with crosstalk between intensity and phase information.^19^ The proposed AA-P algorithm had the best reconstruction quality, as shown in Figs. 4(e1) and 4(e2), compared to the others and showed higher robustness to hybrid aberrations of the optical system owing to the improved optimization strategy of spectral and optical pupil function updates.

Furthermore, by analyzing Table 1 and Figs. 4(a3), 4(d3), and 4(e3) for structural similarity index (SSIM) between the reconstructed phase image of optical pupil function and the simulated wavefront aberration, the proposed AA-P algorithm had a higher match with the SSIM than the EPRY-FPM algorithm. Thus, the phase image of the optical pupil function recovered by the proposed AA-P algorithm could better characterize the wavefront aberration of the FPM optical system. The convergence performance and reconstruction quality results of the four algorithms were further quantified and analyzed by comparing the root mean square error (RMSE) curves of the HR amplitude and phase intensity images reconstructed by the four algorithms under different iterations, and the RMSE values after iterative stable convergence were recorded in Table 2. As shown in Figs. 4(f) and 4(g), the AP algorithm was severely disturbed by the aberration, with the worst reconstruction quality, so it could not converge in normal iterations; the AA algorithm converged normally but its reconstruction quality still needs to be improved; although the EPRY-FPM algorithm corrected the aberration to some extent, comparatively, the AA-P algorithm converged faster and better reconstructed the HR amplitude and phase images, with an improved reconstruction quality of 82.6% and 54.6%, respectively.

**Table 2:** RMSE values of reconstructed intensity and phase for the four algorithms under iterative stable convergence conditions, and SSIM values of the phase of the optical pupil function compared to the simulated wavefront.

Algorithms	Reconstructed amplitude	Reconstructed phase	Phase of pupil
	RMSE ($\times 10^{-1}$)		SSIM
AP	9.107	8.804	—
AA	8.026	7.175	—
EPRY-FPM	5.318	1.876	0.982
AA-P	0.927	0.851	0.997

### 3.2 Real Experiment

**Fig. 5:**
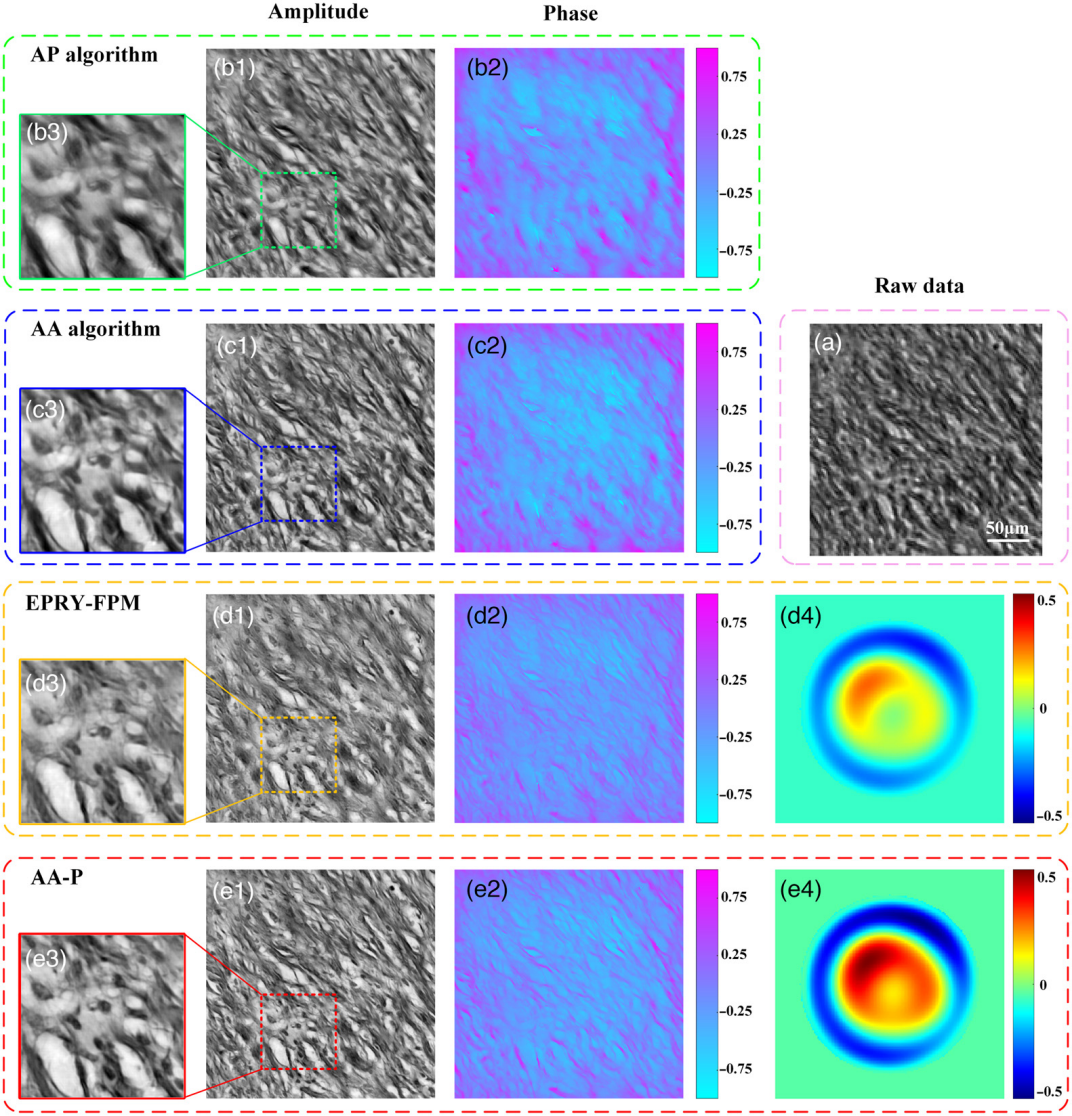
Comparison of reconstruction results using biological sample of HE pathological tissue. (a) LR image captured under central positive incidence of LED illumination. (b) and (c) Amplitude and phase reconstruction results using the AP and AA algorithms, respectively. (d) and (e) Amplitude, phase, and phase of pupil function reconstructed by EPRY-FPM and AA-P algorithm, respectively.

The effectiveness of the proposed AA-P algorithm was verified through simulations. Furthermore, the algorithm was validated on the experimental data of open-source biological samples provided by Zheng et al. to verify its performance.^20^ Experimental parameters in the experiment were set similar to that in the simulation experiment of Sec. 3.1. A biological sample of HE pathological tissue section was chosen, for which 225 LR images were captured under a 15×15 green LED array illumination (central wavelength is 531 nm). Consistent with the simulation experiments, the LR image dataset was reconstructed in the presence of aberrations in an unknown imaging system. The LR image captured by the central LED array illumination was upsampled as the initial HR spectrum shown in Fig. 5(a), and the amplitude and phase images reconstructed by four FPM algorithms were compared. As shown in Figs. 5(b1)–5(b3), the reconstruction quality with the AP algorithm was the worst, with blurred and cloudy reconstructed amplitude and phase intensity image information, respectively. Figures 5(c1)–5(c3) show the HR images reconstructed using the AA algorithm. Comparatively, the clarity of the reconstructed amplitude intensity image by the AA algorithm was significantly improved, with distinct line contours but partially blended and some crosstalk in the recovery of phase information. Figures 5(d1)–5(d3) show the reconstruction results of the EPRY-FPM algorithm, where the phase image reconstruction quality was significantly improved. However, due to its optimization strategy, the lines of the phase image were not clear and distinct and the quality of the reconstructed amplitude image was inferior to that of the AA algorithm, with problems of low overall contrast and blurred local details. Conversely, the HR pathological tissue sections reconstructed by the proposed AA-P algorithm was identified in more detail, with clearly defined tissue lines and higher overall contrast, as shown in Figs. 5(e1)–5(e3). In addition, Figs. 5(d4) and 5(e4) show the fitting results of the phase of the optical pupil function recovered by the EPRY-FPM and AA-P algorithms, respectively. Combined with the simulation experiment results, the proposed AA-P algorithm can be used to further estimate the wavefront aberration of the FPM optical system accurately in real experiments.

To further evaluate the imaging performance of the four algorithms, we compared the RMSE, number of iterations, final error, and run time of their reconstruction results were compared, and the results are listed in Table 3. The RMSE and iterative final error metrics show that the amplitude and phase images reconstructed using the AA-P algorithm had better quality. In addition, the AA-P algorithm had the least number of iterations and required less time, indicating a better convergence performance.

**Table 3:** Comparison of RMSE, number of iterations, final error, and run time of the AP, AA, EPRY-FPM, and AA-P algorithms.

Algorithms	AP		AA		EPRY-FPM		AA-P	
Reconstructed image	Amplitude	Phase	Amplitude	Phase	Amplitude	Phase	Amplitude	Phase
RMSE ($\times 10^{-1}$)	2.319	1.841	1.581	1.822	1.889	1.030	1.439	0.843
Iteration (times)	15		15		12		10	
Final error	3.3825		1.4607		1.2097		1.0870	
Run time (s)	22.02		21.33		20.00		17.14	

**Fig. 6:**
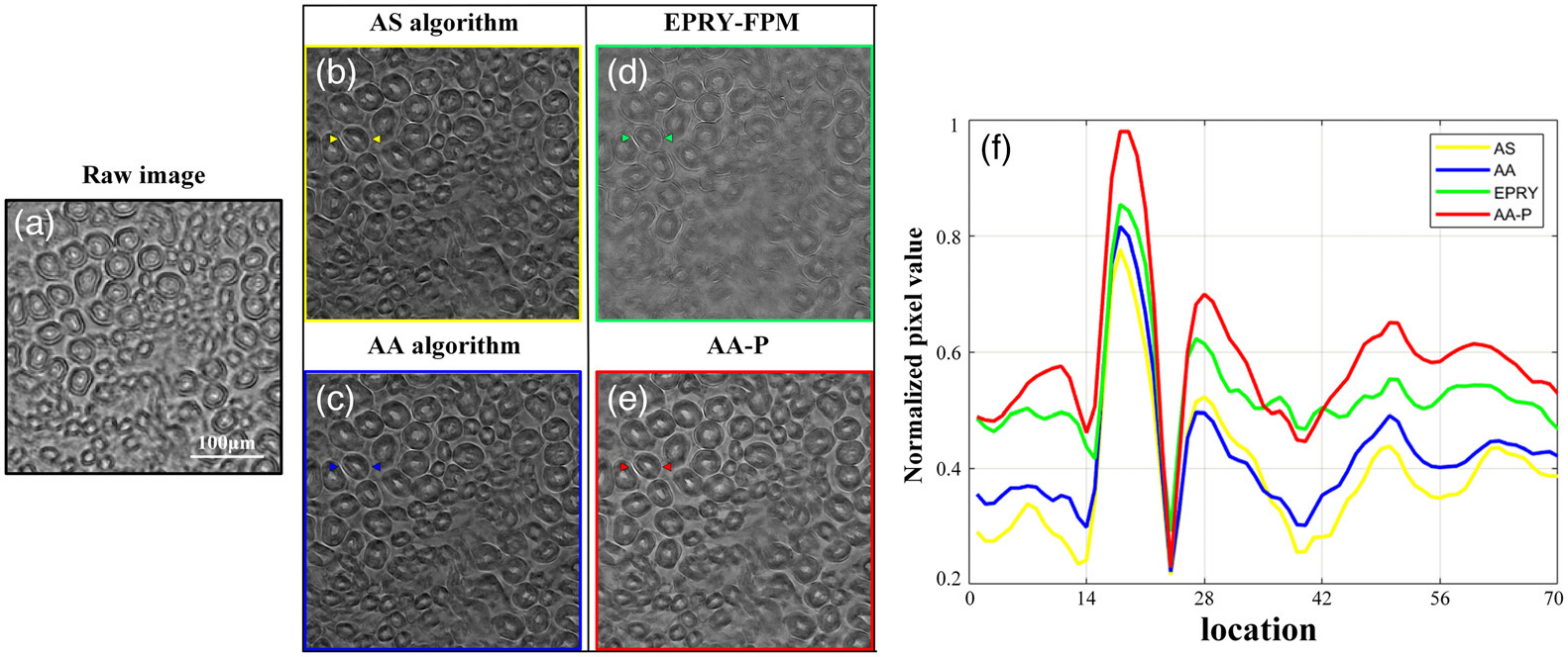
Comparison of reconstruction results using biological sample of the MouseKidney. (a) LR image captured under central positive incidence of LED illumination. (b)–(e) Reconstructed intensity images using AS, AA, EPRY-FPM, and AA-P algorithms. (f) Curve trends of normalized pixel values at the annotation regions for the four reconstruction results.

To verify the stability of the AA-P algorithm for experimental data, additional biological samples were recovered from the FPM optical system. A reconstruction recovery on the kidney fibroblasts (MouseKidney) of a mouse was performed using the same experimental parameters used in the experiment of Sec. 3.2, as shown in Fig. 6. Raw image of a selected region is shown in Fig. 6(a). Figures 6(b) and 6(c) represent the intensity images reconstructed using an adaptive step reconstruction strategy^21^ (AS algorithm) and the AA algorithm, respectively, where the background was blended with the cells, and some distortions were observed when compared to Fig. 6(a), which illustrates that the presence of aberrations can have a serious impact on the fidelity of the FPM reconstructed images. Figure 6(d) shows the intensity image reconstructed using the EPRY-FPM algorithm, which effectively improved the problem of background turbidity, but the overall contrast was not very high. The cells appeared blurry and the reconstruction quality was still unsatisfactory. Comparatively, the reconstructed intensity image of the proposed AA-P algorithm, shown in Fig. 6(e), had a clearer background and cell separation with clean image details, and showed the best reconstruction quality. Furthermore, the curve trends of the normalized pixel values in the annotated regions of Figs. 6(b)–6(e) were compared and the results are shown in Fig. 6(f). The four colors represent the normalized curve trends at the annotation regions for the intensity images reconstructed using the four algorithms. On analyzing the curve trends, it can be concluded that the red curve has the highest upper and lower drops, with a highly obvious concavity and a high average value. Thus, the image reconstructed by the AA-P algorithm had a higher contrast, clearer cell outlines distinguished with more details, and a maximum fidelity to the original image. Thus, the feasibility of the proposed AA-P algorithm for quantitative phase imaging of different samples is further validated.

**Fig. 7:**
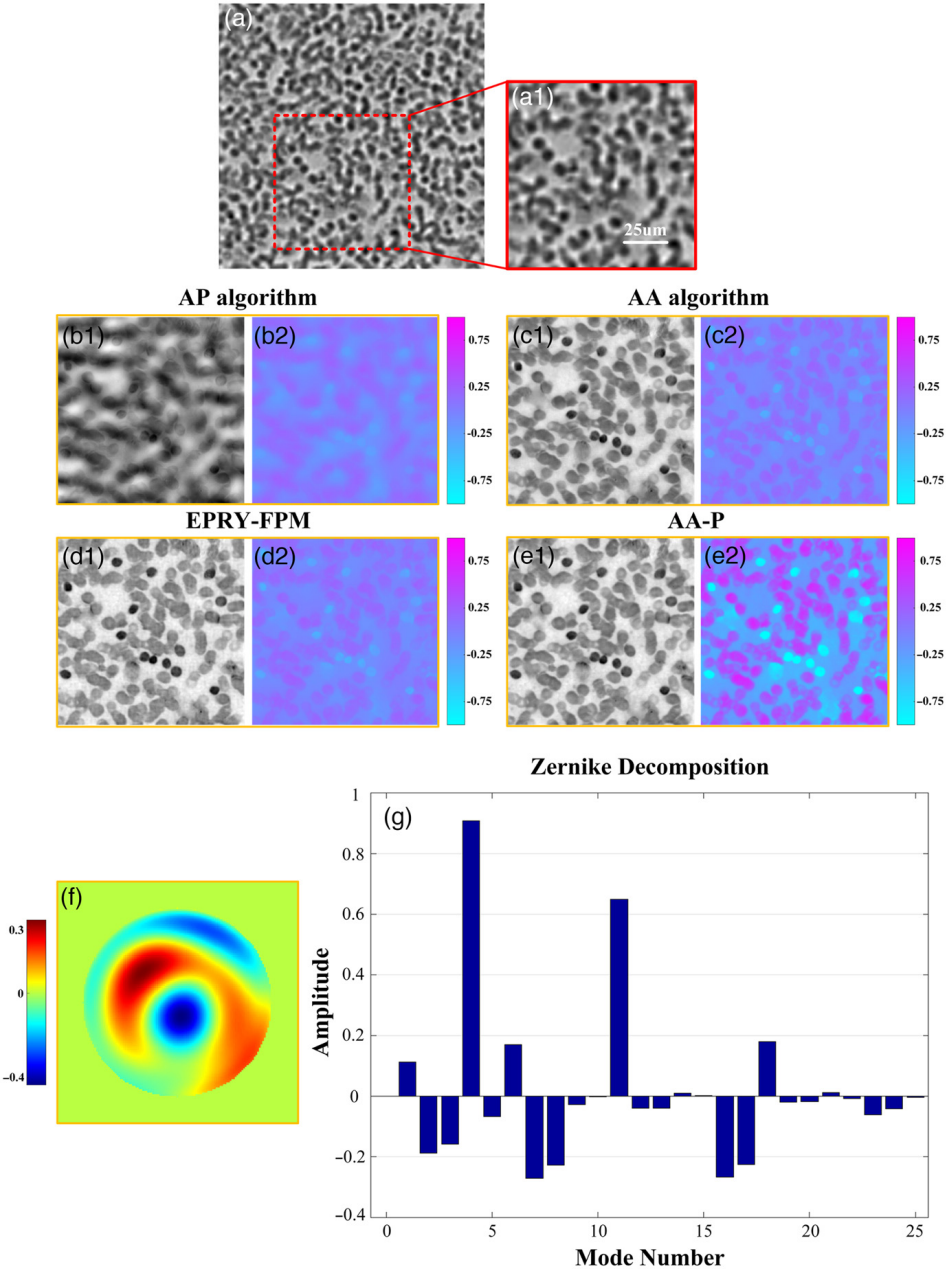
Comparison of reconstruction results using human blood smear biological samples. (a) LR image taken under the central normal incidence of LED illumination. (b)–(e) Amplitude and phase reconstruction results using AP, AA, EPRY-FPM and AA-P algorithms respectively. (For example, b1 is the reconstructed amplitude image, b2 is the reconstructed phase image.) (f) Phase of pupil function reconstructed by AA-P algorithm. (g) Zernike decomposition of pupil function phase, the amplitude of the lowest 25 modes are shown.

Finally, we performed experiments under other optical systems, and we chose to perform reconstruction recovery on human blood smears. 225 LR images were taken using a central 15×15 blue LED array under illumination (central wavelength of 475 nm), and other experimental parameters were consistent with previous experiments. The original images of the selected areas are shown in Fig. 7(a1). Similarly, we compared the reconstructed amplitude and phase results of the four algorithms (AP algorithm, AA algorithm, EPRY-FPM algorithm, and the AA-P algorithm proposed in this paper), and each algorithm was iterated to the convergence condition. Figures 7(b1) and 7(b2) show the reconstructed amplitude and phase images of the AP algorithm, which could not converge to obtain clear reconstruction results and produced serious distortion. Figures 7(c1), 7(c2), 7(d1), and 7(d2) are reconstructed using the AA algorithm and the EPRY-FPM algorithm. Although the amplitude reconstruction results have been greatly improved, the phase reconstruction results have lower contrast and the amplitude reconstruction still has some blurring. However, compared with the previous three algorithms, the amplitude and phase images reconstructed by the AA-P algorithm are better recovered, with clear cell contours and high phase contrast, without image distortion and crosstalk, which further verifies the stability and effectiveness of the AA-P algorithm in recovering different samples under different aberration conditions.

Figure 7(f) shows the fitting results of the phase of the optical pupil function reconstructed by the AA-P method, which can reflect the wavefront aberration of the system, for which we performed the Zernike polynomial decomposition of the first 25 terms, as shown in Fig. 7(g). The analysis shows that the wavefront aberration of the system mainly comes from the tilt of modes 2 and 3, the defocus of mode 4, the astigmatism of modes 5 and 6, and the coma of modes 7 and 8, in addition to which the higher-order aberrations such as spherical, primary trefoil, and tetrafoil are still not negligible.

## 4 Conclusion and Discussion

In this work, we propose an FPM aberration-correction reconstruction framework based on an improved phase-recovery strategy, referred to as the AA-P algorithm, which aims to improve the reconstruction quality of FPM. By optimizing the spectral function and optical pupil function update strategy, the algorithm effectively improves the iterative reconstruction quality and reduces the impact of hybrid wavefront aberration on the FPM reconstruction quality and occurrence of errors in the reconstruction process. The reconstruction quality of the proposed AA-P algorithm is better than the most advanced reconstruction algorithm (AA algorithm) and aberration correction method (EPRY-FPM), and had higher robustness to hybrid aberrations and convergence performance, as demonstrated in experiments with biological samples such as HE pathological tissue, MouseKidney and human blood smears. The strict requirement of wavefront aberration for FPM is further relaxed. In future, the AA-P algorithm can be used for more advanced application scenarios. For example, it is used for dynamic aberration correction of the observed live sample for high quality dynamic high-throughput quantitative phase imaging. This requires future work on improving the imaging efficiency of FPM.

## Code, Data, and Materials Availability

We sincerely acknowledge the open source data set of the biological sample provided by Zheng et al.^20^
